# Effect of maternal vitamin and mineral restrictions on the body fat content and adipocytokine levels of WNIN rat offspring

**DOI:** 10.1186/1743-7075-4-21

**Published:** 2007-10-15

**Authors:** Venu Lagishetty, Vijaya Bhanu Nandiwada, Rajender Rao Kalashikam, Raghunath Manchala

**Affiliations:** 1 Division of Endocrinology and Metabolism, National Institute of Nutrition, Hyderabad – 500 007 India; 2 Division of Neonatology & Developmental Biology and the Neonatal Research Center, Department of Pediatrics, David Geffen School of Medicine UCLA, Los Angeles, CA, 90095, USA

## Abstract

**Background:**

Intrauterine growth retardation due to maternal under-nutrition increases susceptibility to obesity and insulin resistance. We reported earlier in the offspring of mineral or vitamin restricted rat dams, a high body fat percentage and decreased insulin secretion to glucose challenge. This study determined whether or not central adiposity and altered adipocytokine profile were associated with high body fat content.

**Methods:**

Body fat percentage; glucose, insulin and adipocytokine levels in fasting plasma and fresh weights of epididymal fat pads were determined in the six months old male offspring of Wistar NIN rat dams on chronic 50 percent restriction of vitamins or minerals throughout their growth, gestation, lactation and weaned on to restricted diets or restricted mothers/offspring rehabilitated from different time points.

**Results:**

In line with high body fat percent, chronic restriction of vitamins and minerals increased the epididymal fat pad weight. Maternal vitamin restriction decreased plasma adiponectin and increased leptin levels whereas mineral restriction decreased both. Both the treatments did not affect plasma TNF-α levels or insulin resistance status (HOMA-IR). Rehabilitation from parturition but not weaning, rescued the changes in the offspring.

**Conclusion:**

High body fat percentage in the offspring of vitamin restricted or mineral restricted rat dams was associated with increased abdominal adiposity (epididymal fat pad weight) and differential expression of adipocytokines but not insulin resistance. The changes could be mitigated by rehabilitation from birth but not weaning.

## Background

Maternal under-nutrition impairs intrauterine development and increases adiposity, insulin resistance (IR) and associated metabolic disturbances in the later life of the offspring [1]. We reported earlier that chronic 50 percent restriction of minerals (MR) or vitamins (VR) in Wistar NIN (WNIN) rat dams increased the body fat percentage in offspring and decreased their insulin response to glucose challenge [2,3]. We also reported that increased oxidative stress and impaired antioxidant status were associated with maternal VR but not MR induced changes in offspring [2,3]. We now report whether or not increased body adiposity in the VR and MR offspring is associated with increased abdominal adiposity, altered expression of adipocytokines and insulin resistance.

Adipocytokines, the adipocyte derived bioactive molecules, mediate the systemic effects of obesity on health and regulate lipid metabolism as well as IR [4]. In fact the adipocytokine: leptin is important in the pathogenesis of eating disorders and obesity and mediates the neuro-endocrine response to food deprivation [5]. Overproduction of TNF-α modulates IR in obesity [6]. Furthermore, reduced expression of adiponectin and low plasma adiponectin levels are implicated in the pathogenesis of obesity and type 2 diabetes [7]. Indeed, mice lacking adiponectin display IR in some conditions [8,9]. Considering that maternal VR and MR increased body adiposity and impaired glucose stimulated insulin secretion in the offspring, we have determined whether or not altered expression of adipocytokines was associated with these changes.

## Methods

### Experimental Animals

All animal experimental procedures were carried out in accordance with the 'principles of laboratory animal care' (NIH publication no. 85-23, revised 1985) and with the approval of the "Institute's ethical committee on animal experiments" at National Institute of Nutrition, Hyderabad, India.

Female, weaning WNIN rats obtained from National Centre for Laboratory Animal Sciences, National Institute of Nutrition, Hyderabad, India were used in these experiments. The protocol used for animal grouping, feeding, breeding and maintenance was described by us previously [2,3]. Briefly, the rats received for 12 weeks, a control (AIN 93G) diet or a similar diet with 50 percent (of control diet) restriction of mineral/vitamin mixture, mated with control males and continued on their respective diets through out gestation. At parturition, a third of the restricted dams were shifted to control diet while the remaining continued on restricted diets. Half the number of pups born to these restricted dams were weaned on to control diet while the other half continued on the respective restricted diet.

Blood was collected from the offspring (at six moths of age) after an overnight fast and used for the determination of plasma glucose, insulin and adipocytokines. Insulin resistance (HOMA IR) was computed and the body fat of the animals content was determined by the TOBEC method as described by us earlier [2,3]. The animals were sacrificed by carbon dioxide inhalation, epididymal fad pads were excised quickly and their fresh weight determined.

### Adipocytokine levels

Plasma leptin and TNF – α concentrations were determined using a rat specific enzyme linked immunosorbent assay (R&D Systems, MN, USA). Rat specific RIA kit (Linco Research, MO, USA) was used to determine plasma adiponectin levels. The lower limits of detection were less than 22 pg/mL for leptin; 5 pg/mL for TNF-α and 1 ng/mL for adiponectin.

### Statistical analysis

Data was subjected to statistical analysis using SPSS package (version 10.0) and values presented as mean ± SEM. Data was analyzed by one-way analysis of variance (ANOVA) followed by Post Hoc least significant difference (LSD) test. Wherever the heterogeneity was observed in the variance, differences between groups were tested by the non-parametric Mann – Whitney U test. The differences were considered significant only if *p *< 0.05.

## Results

### Body fat content, epididymal fat pad weight and insulin resistance

In line with the high body fat percentage observed in the offspring of VR and MR rat dams, the fresh weight of the epididymal fat pads was significantly higher in them (compared to controls) at six months of age (Table 1 &2). Rehabilitating VR mothers from parturition and their offspring from weaning (VSP) but not weaning VR offspring to control diet (VSW) reversed the body fat percentage and epididymal fat pad weight to levels comparable to controls (Table 1). While both the rehabilitation regimes mitigated the maternal MR induced increase in body fat percentage of the offspring only partially, MSP but not MSW could rescue the increased weight of the epididymal fat pad (Table 2).

**Table 1:** Body weight, fat content and plasma adipocytokine levels in the offspring of vitamin restricted WNIN rat dams on postnatal day 180

	**VC**	**VR**	**VSP**	**VSW**
Body weight (g)	362 ± 6.4^a^	346 ± 2.24^b^	392 ± 4.84^a^	351 ± 7.6^a^
Percent of body fat	16.8 ± 0.57^a^	19.9 ± 0.72^b^	17.2 ± 0.63^a^	18.7 ± 0.61^b^
Epididymal fat pad weight (g/100 g bodyweight)	1.52 ± 0.04^a^	1.98 ± 0.04^b^	1.69 ± 0.09^a^	1.92 ± 0.13^b^

**Adipocytokine levels**				

Adiponectin (μg/ml)	3.04 ± 0.08^a^	2.21 ± 0.31^b^	3.21 ± 0.36^a^	2.34 ± 0.27^b^
Leptin (ng/ml)	1.78 ± 0.50^a^	4.45 ± 0.65^b^	2.17 ± 0.67^a^	4.61 ± 1.44^b^
TNF – α(pg/ml)	33.6 ± 20.4	51.7 ± 29.6	31.2 ± 26.1	33.9 ± 15.2

**Insulin resistance**				

HOMA-IR	8.44 ± 0.82	10.27 ± 0.98	8.64 ± 0.69	9.01 ± 0.88

*VC*, Control diet through out; *VR*, vitamin restriction through out; *VSP*, rehabilitation of restricted mothers from parturition and their pups from weaning; and *VSW*, vitamin restricted offspring weaned on to control diet. Values are mean ± SE (n = 6). Means without a common letter are significantly different by one way ANOVA (*p *< 0.05) followed by post hoc LSD test.

**Table 2:** Body weight, fat content and plasma adipocytokine levels in the offspring of mineral restricted WNIN rat dams on postnatal day 180

	**MC**	**MR**	**MSP**	**MSW**
Body weight (g)	354 ± 6.56^a^	326 ± 6.44^b^	316 ± 4.31^b^	316 ± 6.62^b^
Percent body fat	13.3 ± 0.23^a^	16.9 ± 1.13^b^	15.9 ± 0.80^ab^	15.7 ± 0.40^ab^
Epididymal fat pad weight (g/100 g bodyweight)	1.0 ± 0.10^a^	1.8 ± 0.12^b^	1.5 ± 0.09^a^	1.9 ± 0.06^b^

**Adipocytokine levels**				

Adiponectin (μg/ml)	3.30 ± 0.17^a^	2.69 ± 0.38^b^	3.15 ± 0.27^a^	2.17 ± 0.19^b^
Leptin (ng/ml)	1.65 ± 0.05^a^	0.96 ± 0.13^b^	1.69 ± 0.42^a^	1.05 ± 0.08^b^
TNF – α(pg/ml)	21.5 ± 9.6	43.5 ± 19.5	38.5 ± 22.4	65.2 ± 24.4

**Insulin resistance**				

HOMA-IR	14.0 ± 2.38	10.0 ± 1.2	8.71 ± 1.51	8.29 ± 0.71

*MC*, Control diet through out; *MR*, mineral restriction through out; *MSP*, rehabilitation of restricted mothers from parturition and their pups from weaning; and *MSW*, mineral restricted offspring weaned on to control diet. Values are mean ± SE (n = 6). Means without a common letter are significantly different by one way ANOVA (*p *< 0.05) followed by post hoc LSD test.

Despite their significant effects on the body fat percentage and epididymal fat pad weight in the offspring, neither maternal VR nor MR had any effect on their IR as assessed by HOMA IR (Table 1 &2). As a corollary, the two rehabilitation regimes had no effect on this parameter.

### Effect of maternal vitamin restriction on adipocytokine levels

Plasma adiponectin levels were significantly decreased (p < 0.05) and leptin levels increased (p < 0.05) in VR offspring (Table 1) compared to the control (VC) offspring on post natal day 180. However plasma TNF-α levels were comparable among different groups of the offspring. In line with the effects seen on the increased percentage of body fat and fresh weight of epididymal fat pads, VSP but not VSW corrected the changes in adiponectin and leptin levels by six months of age (Table 1).

### Effect of maternal mineral restriction on adipocytokine levels

Chronic MR in WNIN rat dams decreased both plasma adiponectin and leptin levels significantly (p < 0.05) in the offspring (Table 2) compared to controls (MC). Similar to the observations made in the offspring of rehabilitated VR dams, MSP but not MSW corrected these changes. Plasma TNF-α levels were comparable among the different groups of the offspring (Table 2).

## Discussion

Increased body adiposity and/or altered lipid metabolism not only precede but also lead to tissue insulin resistance [10,11]. Inline with these reports, the offspring of both VR and MR rat dams had higher body fat percentage compared to controls. The increase in fresh weight of the epididymal fat pads suggests that the increased body fat content in the VR and MR offspring could be due to an increase in central adiposity, a hall mark feature associated with and predisposes individuals to IR later in life [11].

Similar to their effects on body fat percentage, rehabilitation of VR dams from parturition and their offspring from weaning but not weaning VR offspring to control diet rescued the increased epididymal fat pad weight. This observation stresses the importance of vitamin nutrition during lactation in programming the body composition of the offspring and is in line with our similar findings earlier [2]. That both the rehabilitation regimes mitigated the maternal MR induced increase in body fat percentage of the offspring only partially suggests its irreversibility to a great extent as compared to that induced by maternal VR. This is corroborated by the observation that even the increased weight of epididymal fat pads was corrected only partly by MSP but not MSW.

The significant decrease in plasma adiponectin and increase in leptin levels seen in VR offspring are in agreement with earlier reports which showed that similar changes were associated with increased body fat and IR [5,7]. Although increased leptin levels are usually associated with increased food intake, food intake was not increased in the VR offspring suggesting that they were probably leptin resistant. These results in the VR offspring suggest an association between the altered expression of adiponectin and leptin and their high body adiposity, albeit their causal relationship remains to be delineated.

The decreased plasma adiponectin levels seen in MR offspring are in agreement with similar reports earlier [7] and corroborate the increased percentage of body fat observed in them. However, our observation that hypoleptinemia was associated with high body fat percentage in MR offspring is at variance with many earlier studies demonstrating an association between high plasma leptin levels and high percentage of body fat [5]. Further studies are clearly needed to delineate the role if any of the hypoleptinemia in maternal MR induced increase in body fat percentage in the offspring. Interestingly, hypoleptinemia observed here is in line with leptin deficiency reported in the genetically obese rodent models [12,13] and also with the hypoleptinemia reported in type 1 and 2 diabetic patients [14].

Increased TNF-α levels are associated with increased adiposity and IR [6]. However chronic MR or VR did not alter plasma TNF-α levels significantly. This finding appears to rule out a role for TNF-α in maternal VR and MR induced changes in the body fat of the offspring.

That rehabilitation of VR and MR dams from parturition but not weaning the VR and MR pups to control diet could mitigate the changes in adipocytokine levels stresses the importance of vitamin and mineral nutrition during lactation in modulating adipocytokine expression in addition to the body adiposity of the offspring. However the finding that despite comparable (to controls) leptin levels, MSP offspring had higher percentage of body fat and epididymal fat pad weight is perplexing and suggests that hypoleptinemia and leptin resistance may both be involved in maternal MR induced changes in adiposity of the offspring.

Not withstanding the effects seen in the body fat percentage, epididymal fat pad weight and plasma adipocytokine levels, neither maternal VR nor MR had any significant effect on the IR status in the offspring as assessed by the HOMA IR values. Lack of any effect on the IR status of the offspring could be due to the shorter duration of VR/MR and/or the lower magnitude of VR/MR employed in these studies. Considering our earlier reports [2,3] that maternal VR/MR irreversibly decreased insulin secretion by the offspring to a glucose challenge, the increased body fat percentage observed here in the VR/MR offspring suggests that maternal VR/MR could lead to a hyperglycemic state in the offspring at a later age. That the rehabilitation regimes had similar effects on plasma adipocytokines, body fat percentage and glucose stimulated insulin suggests that adipocytokines play an important role in maternal VR/MR induced programming of glucose stimulated insulin secretion and hence glucose metabolism in the offspring in addition to their body adiposity.

## Conclusion

The present observations indicate that increased central adiposity underlies the increased percentage of body fat in the offspring of VR and MR rat dams. Altered expression of adiponectin and leptin is associated with maternal VR and MR induced changes in the body adiposity (composition) of the offspring but maternal VR/MR differentially modulate their expression. That rehabilitation of restricted mothers from parturition but not weaning the restricted offspring to control diet could correct the changes in adipocytokine levels, epididymal fat pad eight and body fat percent may suggest a causal relationship, which however needs to be established. The results also suggest the importance of vitamin and mineral nutrition during lactation in modulating the body adiposity of the offspring, specially the central adiposity, a fore runner for IR and associated diseases in their later life.

## Abbreviations

MC: control diet through out

MR: mineral restriction through out

MSP: rehabilitation of mineral restricted mothers from parturition and their pups from weaning

MSW: mineral restricted offspring weaned on to control diet.

VC: control diet through out

VR: vitamin restriction through out

VSP: rehabilitation of vitamin restricted mothers from parturition and their pups from weaning

VSW: vitamin restricted offspring weaned on to control diet.

WNIN: Wistar NIN

## Competing interests

The author(s) declare that they have no competing interests.

## Authors' contributions

VL participated in the conception and design of the study, data collection, tissue sampling, statistical analysis and drafting of the manuscript.

VBN participated in animal experimentation and tissue sampling.

RRK participated in animal experimentation and carried out the immunoassays.

RM conceived of the study, and participated in its design, coordination and drafting of the manuscript.

All authors read and approved the final manuscript.
